# Mass‐ratio and complementarity effects simultaneously drive aboveground biomass in temperate *Quercus* forests through stand structure

**DOI:** 10.1002/ece3.8312

**Published:** 2021-11-12

**Authors:** Wen‐Qiang Gao, Xiang‐Dong Lei, Dong‐Li Gao, Yu‐Tang Li

**Affiliations:** ^1^ Key Laboratory of Forest Management and Growth Modelling State Forestry and Grassland Administration Institute of Forest Resource Information Techniques Chinese Academy of Forestry Beijing China; ^2^ Planning and Design Institute of the Forest Products Industry of the State Forestry and Grassland Administration Beijing China; ^3^ Jilin Forestry Inventory and Planning Institute Changchun China

**Keywords:** complementarity effect, functional composition, functional diversity, mass‐ratio effect, phylogenetic diversity, species richness

## Abstract

Forests play a key role in regulating the global carbon cycle, a substantial portion of which is stored in aboveground biomass (AGB). It is well understood that biodiversity can increase the biomass through complementarity and mass‐ratio effects, and the contribution of environmental factors and stand structure attributes to AGB was also observed. However, the relative influence of these factors in determining the AGB of *Quercus* forests remains poorly understood. Using a large dataset retrieved from 523 permanent forest inventory plots across Northeast China, we examined the effects of integrated multiple tree species diversity components (i.e., species richness, functional, and phylogenetic diversity), functional traits composition, environmental factors (climate and soil), stand age, and structure attributes (stand density, tree size diversity) on AGB based on structural equation models. We found that species richness and phylogenetic diversity both were not correlated with AGB. However, functional diversity positively affected AGB via an indirect effect in line with the complementarity effect. Moreover, the community‐weighted mean of specific leaf area and height increased AGB directly and indirectly, respectively; demonstrating the mass‐ratio effect. Furthermore, stand age, density, and tree size diversity were more important modulators of AGB than biodiversity. Our study highlights that biodiversity–AGB interaction is dependent on the regulation of stand structure that can be even more important for maintaining high biomass than biodiversity in temperate *Quercus* forests.

## Graphical abstract

This study demonstrates that tree species diversity, functional trait composition, stand age, structure (i.e., stand density and tree size diversity), and environmental factors contribute to the geographic variation in aboveground biomass (AGB) of temperate *Quercus* forests. We concluded that biodiversity–AGB interaction is dependent on the regulation of stand structure that can be even more important for maintaining high biomass than biodiversity in temperate *Quercus* forests.

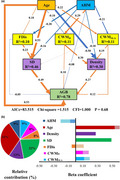

## 1 INTRODUCTION

Forests play important roles in terrestrial ecosystems as the most important biodiversity repositories and components of the global carbon cycle (Houghton et al., 2009; King et al., 2012). Variations in the biodiversity, as well as structural and abiotic factors (e.g., climate and soil), determine forest ecosystem functioning (Ali et al., 2019a; Paquette et al., 2015; Prado‐Junior et al., 2016; Vargas‐Larreta et al., 2021; Yuan et al., 2016; Zhang & Chen, 2015). Hence, a better understanding of the relationship between multiple abiotic and biotic factors with aboveground biomass (AGB) is critical to sustaining forest ecosystem functions (Huang et al., 2018; Yuan et al., 2018). However, underlying mechanisms associated with this relationship have still not well understood.

Biodiversity can increase the AGB (Luo et al., 2019; Yuan et al., 2016; Zhang & Chen, 2015). Two hypotheses proposed to explain the positive effect of biodiversity on AGB are complementarity and mass‐ratio effects. The complementarity effect predicts that increasing biodiversity can increase resource‐use efficiency. Therefore, increased biodiversity enhances productivity (Tilman et al., 1997) because dissimilar species provide unique contributions to ecosystem function (Barry et al., 2019; Cadotte, 2017). Besides species richness (Mouquet et al., 2002; Tilman et al., 1997), recent studies have found that functional and/or phylogenetic diversity can have greater explanatory power on AGB than species richness (Díaz & Cabido, 2001; Flynn et al., 2011; Fotis et al., 2018; Ruiz‐Benito et al., 2014). Functional diversity can better capture the degree of functional redundancy and niche overlap. Phylogenetic diversity contains information on evolutionary distances, and it is used to reflect the diversity of phylogenetically conserved traits related to resource capture, use, and storage (Faith, 1992; Satdichanh et al., 2019). Therefore, the positive effect of species richness, functional, and phylogenetic diversity on AGB can be considered as the complementarity effects. The mass‐ratio effect assumes that variation in AGB is driven by the trait values of the dominant species, which is captured by the community‐weighted mean (CWM) of trait values (Cadotte, 2017). Thus, the positive relationship between the CWM trait values and AGB indicated the mass‐ratio effect (Fotis et al., 2018). These two hypotheses have been deemed to work together in different ecosystems (Cardinale et al., 2007; Fotis et al., 2018; Sonkoly et al., 2019). However, their relative importance is not fully understood. For example, Hao et al. (2020) found that mass‐ratio effects were more important than the complementarity effect in driving the biomass of temperate secondary forests dominated by *Juglans mandshurica*, *Acer mono*, *Tilia amurensis*, *Tilia mandshurica*, *Pinus koraiensis*, *Betula platyphylla*, and *Populus davidiana* in northeastern China. Fotis et al. (2018) found that AGB is driven by mass‐ratio effects, but not complementarity effects, in a temperate deciduous forest dominated primarily by *Acer rubrum*, *Acer saccharum*, and *Liriodendron tulipifera*. These studies further suggest that the biodiversity effect on AGB varied with forest types.

Beyond biodiversity, stand age, and forest structure, such as stand density and tree size complexity, also affect AGB in natural forests (Forrester & Bauhus, 2016; Forrester et al., 2013; Zhang & Chen, 2015). Stand age can enhance AGB via an increase in tree size (Barry et al., 2019; Becknell & Powers, 2014). The higher stand densities are thought to increase AGB through a higher canopy packing (Forrester et al., 2018; Morin, 2015). Diverse structures result in leaf layering and multilayered canopies, and thus increase light capture and use among component species in a community (Lei et al., 2009). The structural diversity has greater explanatory power for biomass and productivity than species richness alone in forest ecosystems (Ali et al., 2019a; Fotis et al., 2018; Park et al., 2019). Importantly, forest communities with richer biodiversity are associated with diverse structures (Ali et al., 2019b) and higher stem density (Chisholm et al., 2013), suggesting that biodiversity can also increase AGB via the stand structure. However, little is known about how stand structure modify complementarity and/or mass‐ratio effects.

Environmental factors are the key regulators of AGB in forests at large scales (Ali et al., 2020; Fotis et al., 2018; Jucker et al., 2016). Previous studies indicated that climate and soil can, directly and indirectly, affect forest biodiversity and AGB (Ali et al., 2019a; Zhang & Chen, 2015). Environmental factors (e.g., climate and soil factors) may influence the growth and distribution of tree species (Matias et al., 2017; Paquette & Messier, 2011), which, in turn, affect the tree species composition and stand structure (Ali et al., 2020; Ouyang et al., 2019; Zhang & Chen, 2015). Therefore, environmental factors should be considered when testing the drivers of AGB in forest ecosystems.

*Quercus* forests are the largest forest component occupying 9.21% and 8.32% of the total forest area and volume in China (State Forestry & Grassland Administration, 2019) and is one of major forest types on earth. Most of them are secondary forests with varied tree species compositions. Understanding the mechanisms driving the AGB of *Quercus* forests is of increasing significance to guide forest management. However, there was a knowledge gap on the drivers of AGB *Quercus* forests, especially the biodiversity–AGB relationships at large scales. Therefore, in this study, our objective was to integrate abiotic (climate and soil) and biotic (biodiversity, stand age, and stand structure) factors to assess the drivers of AGB of *Quercus* forests across Jilin Province in northeast China using a dataset from 523 permanent sample plots. We hypothesized that: (1) Tree species diversity and functional composition will have a positive effect on AGB through mass‐ratio and/or complimentary effects; (2) the effect of complementarity and/or mass‐ratio is dependent on stand structure, because the higher biodiversity is associated with diverse structures and higher stand density (Ali et al., 2019b; Chisholm et al., 2013); (3) abiotic and biotic factors also exert direct and indirect effects on AGB through their effects on biodiversity and stand structure.

## 2 MATERIALS AND METHODS

### 2.1 Study area and forest plots

**FIGURE 1:**
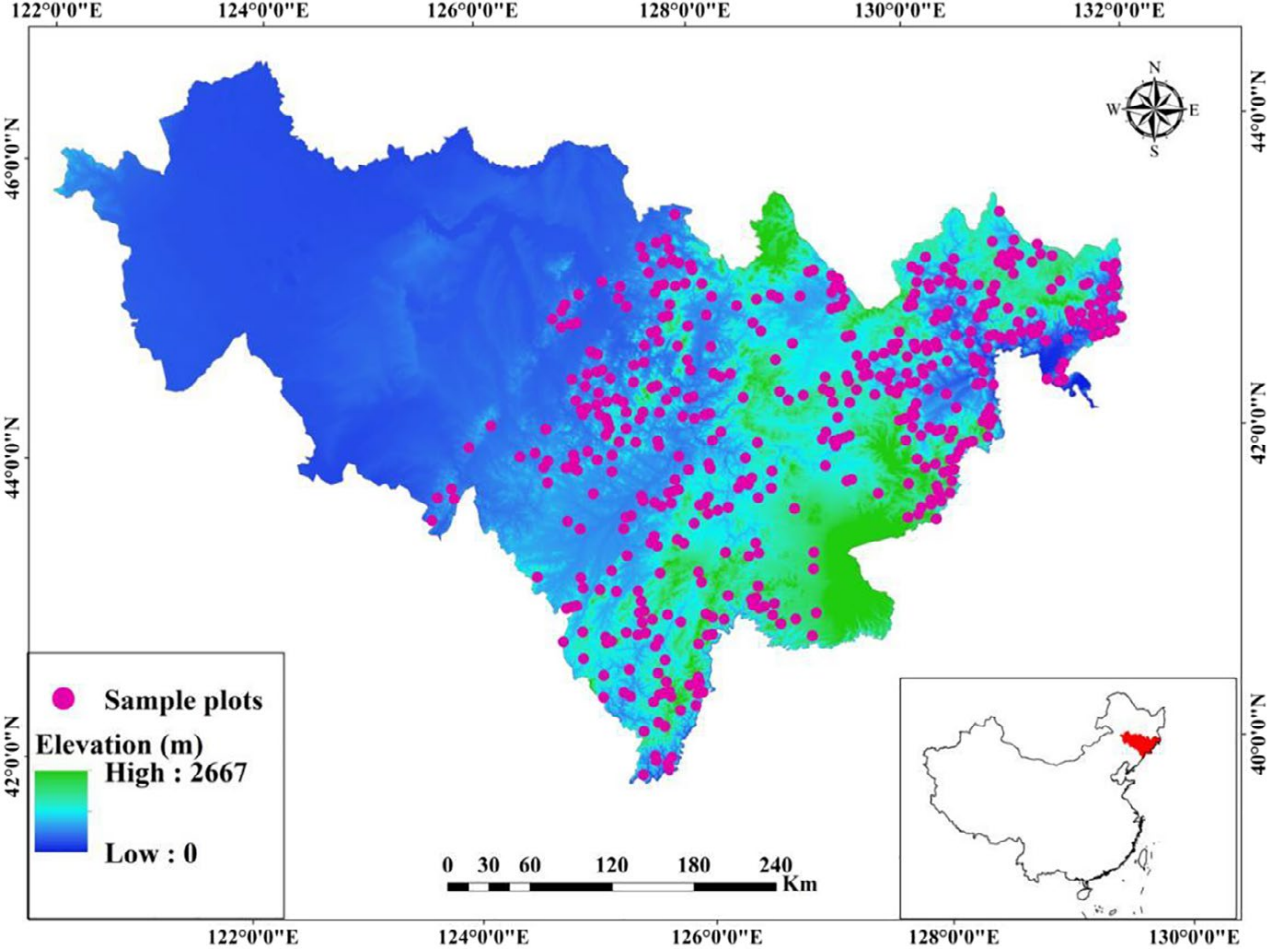
Spatial distribution of the *Quercus* sample plots in Jilin Province, northeast China

The study area was in Jilin Province (40°52′–46°18′N, 121°38′−131°19′E) in northeast China (Figure 1). As one of the most important natural forest regions in China, *Quercus* forests in the province provide both timber and other ecosystem services. The climate, high‐latitude East Asia monsoon, is temperate continental with warm summer, cold winter, abundant precipitation, and a short growing season. The mean annual temperature is 3.9℃, and the mean annual precipitation is 547 mm.

The data of stand characteristics used in this study were retrieved from the permanent sample plots of the 9th National Forest Inventory (NFI) in 2014. Systematic sampling was used with a 4 × 8 km grid across Jilin Province (Figure 1). Each plot was a square with an area of 600 m^2^. We selected plots with the proportion of *Quercus* larger than 30% by basal area, and in total 523 plots with weak human disturbances (the cutting intensity <1% by basal area) (see the spatial distribution of the sample plots in Figure 1). According to the protocols of the National Forest Inventory standards issued by the State Forestry Administration of China, geographic location (latitude and longitude) and altitude, tree species, DBH (1.3 m) of individual trees with DBH ≥ 5 cm, and stand age were recorded. Besides *Quercus mongolica*, other major tree species include *Pterocarya stenoptera*, *Fraxinus mandshurica*, *Picea jezoensis*, *B. platyphylla*, *Populus ussuriensis*, *J. mandshurica*, *Phellodendron amurense*, and *T. amurensis*.

AGB values of all tree species were calculated using DBH‐based allometric equations (Table S1). The total AGB per plot was the sum of the aboveground biomass of all trees with DBH ≥ 5 cm, which was then converted to tons per hectare (t ha^−1^).

### 2.2 Environmental data

Climate variables used in the analysis included mean annual temperature (MAT), mean annual precipitation (MAP), and annual heat–moisture index (AHM). AHM is a biologically relevant indicator of aridity, which was calculated as the ratio of temperature and precipitation (Wang et al., 2012). Based on the geographical location of plots, we extracted climate variables from ClimateAP v2.20 (Wang et al., 2017), and we used the mean values of these climate variables from 1981 to 2010.

Soil variables included soil pH and cation exchange capacity (CEC) representing available soil nutrients for plant growth (Ali et al., 2019a; Poorter et al., 2017). Soil pH and CEC in each plot were derived from the China Dataset of Soil Properties for Land Surface Modelling (Wei et al., 2013). We used the mean values of soil pH and CEC from the first to the fifth layer (0–50 cm) for each plot.

### 2.3 Biodiversity and stand structure

Tree species diversity (including species richness, functional diversity, and phylogenetic diversity) and functional composition were calculated to examine their effects on AGB and elucidate underlying mechanisms. Functional diversity (FDis) represents the difference in functions or characteristics of species in a community (Laliberté & Legendre, 2010). FDis was calculated as the dispersion of functional traits of each plot using the mean trait value of species (Table S1), including specific leaf area (SLA, m^2^/kg), species wood density (WD, g cm^−3^), and maximum tree height (H, m). These functional traits are physical characteristics that affect the growth, survival, and reproduction of individuals, and therefore, the AGB (Garnier et al., 2004). Functional composition is defined as the community weighted mean (CWM) of traits. The community‐weighted mean (CWM) of single traits reflects the relative dominance of species (Garnier et al., 2004), and it was calculated as the mean trait value of a plant in a community. The values of both FDis and CWM of traits were calculated using the dbFD function in the‘FD’ package in R (R Development Core Team, 2013). SLA values were extracted from the literature (Niu et al., 2017; Wang et al., 2018). The wood density values were obtained from the database of global wood density (Zanne et al., 2009). The recorded maximum height of each species was compiled from Flora of China (Editorial Committee of Flora of China, 2004). Phylogenetic diversity was represented as Faith's PD that is the sum of total phylogenetic branch lengths, weighted by abundance (Faith, 1992). The phylogenetic analysis was implemented using Phylocom version 4.2 (Webb et al., 2008).

Stand structure includes stand density and tree size diversity (SD), of which SD was quantified based on the Shannon index of DBH in this study (Eq. [1]) (see Lei et al., 2009).

(1) $$\text{SD}=‐\sum_{i=1}^{d}p_{i}*\log p_{i}$$

where *p_i_* is the relative basal area of the *i*th diameter class in a given plot, and *d* is the number of diameter classes. The diameter class width was set to 2 cm.

### 2.4 Statistical analyses

Aboveground biomass (AGB) was ln‐transformed prior to analyses. All variables of abiotic and biotic were standardized before conducting the analysis. Linear regression analyses were used to examine bivariate relationships between AGB and biodiversity.

Multiple linear mixed‐effects (LME) models were used to examine the effects of biodiversity, stand age, stand structure, soil, and climate variables on the AGB of *Quercus* forests (Eq. (2)). We excluded MAP and phylogenetic diversity from a pair of candidate variables with a correlation coefficient larger than 0.75 to avoid the bias induced by multicollinear variables (Figure S1). The full model included two climate variables (MAT and AHM), two soil variables (soil pH and CEC), two diversity indices (species richness and functional diversity), CWM functional trait values (maximum height, SLA, and wood density), stand age, and two structural variables (stand density and structural diversity). Region (county)‐level random effect was included in the model intercept. Model selection was then conducted by comparing all possible models based on the corrected Akaike information criterion (AICc). For each response variable, we calculated the average model based on selected models (ΔAICc < 2) (Table S2), as implemented in the R package‘MuMIn’ (Bartoń, 2016).

(2) $$\begin{array}{cc} \text{LnAGB} & =\beta_{0}+\beta_{1}*\text{diversity}+\beta_{2}*\text{CWM}+\beta_{3}*\text{structure}\\ & +\beta_{4}*\text{age}+\beta_{5}*\text{climate}+\beta_{6}*\text{soil}+b_{\text{county}}+\varepsilon , \end{array}$$

where AGB is aboveground biomass; diversity is species richness and functional diversity; CWM is the CWM of height, SLA, and wood density; structure represents the tree size diversity and stand density; age is stand mean age; climate and soil are candidate variables mentioned above; *β*_0_ is the estimated fixed intercept; *β*_1_, *β*_2_, *β*_3_, *β*_4_, *β*_5_, and *β*_6_ are the model coefficients estimated for the biodiversity, CWM, structural, age, climate, and soil, respectively; *b*_county_ represents the random effect; and ε represents the error term.

**FIGURE 2:**
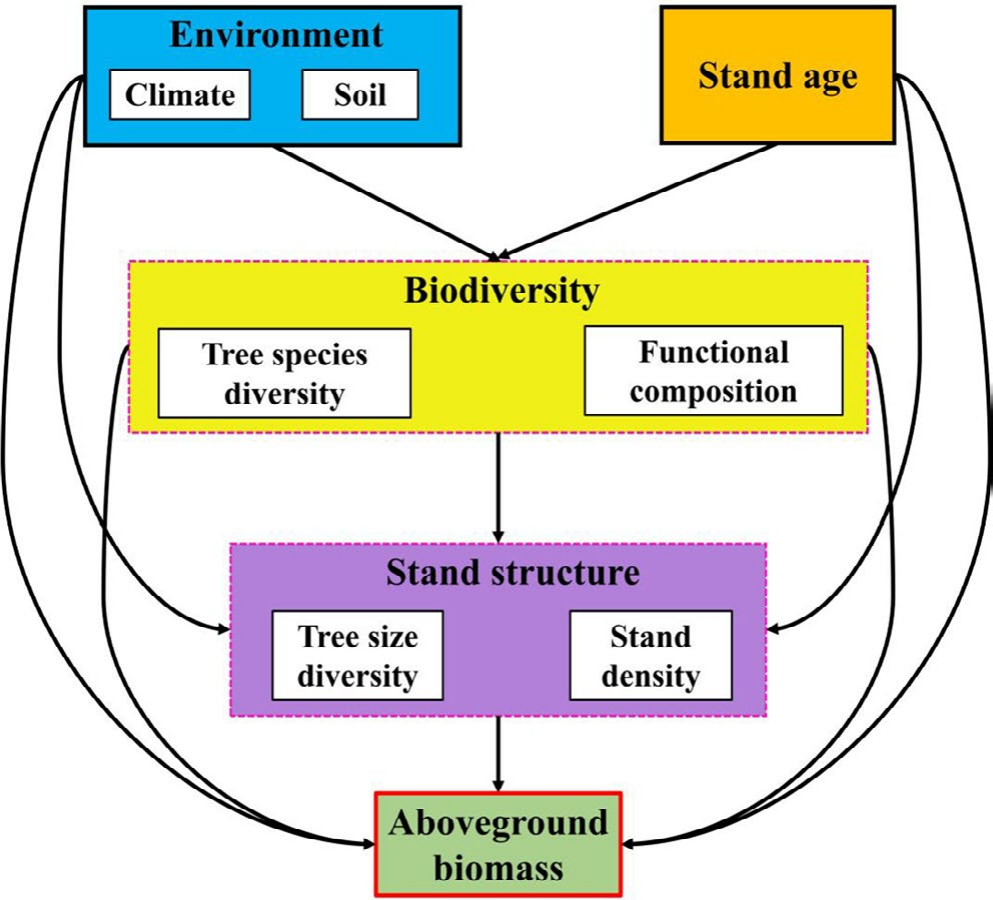
Hypothetical causal model for structural equation model (SEM) exploring the effects of biodiversity (species richness, functional diversity, phylogenetic diversity, functional trait composition), stand age, structure, and environmental variables on aboveground biomass (AGB). We predict that environment, stand age, and biodiversity directly or indirectly affect AGB through altering stand structural attributes

Structural equation modeling (SEM) was performed to test the direct and indirect effects of the driving factors above‐mentioned on AGB based on our conceptual model (Figure 2). The best‐fit SEM was evaluated based on a non‐significant Chi‐square (*χ* ^2^) test statistic (*p* > .05), comparative fit index (CFI) > 0.95, and lowest AIC value (Tables S3 and S4). We only reported the results derived from the selected best‐fitted SEM. The relative contribution of each predictor to AGB was calculated as the ratio between the beta coefficient of a given predictor and the sum of beta coefficients of all predictors and expressed as a percentage. We used the total standardized effect and beta coefficient (i.e., direct and indirect effects) of a given predictor to maintain consistency between our conceptual model (Figure 2) and tested SEMs (Yuan et al., 2019). The SEM model was constructed using the AMOS software (IBM SPSS Amos v24).

## 3 RESULTS

### 3.1 Effects of biodiversity on aboveground biomass

When bivariate relationships between biodiversity and AGB were examined, AGB was positively correlated with functional diversity (Table 1; *R* ^2^ = .021, *p* < .001), but not significantly correlated with species richness and phylogenetic diversity (Table 1, *p* > .05). Regarding the functional composition, AGB was positively correlated with CWM of SLA (CWM_SLA_) (Table1; *R* ^2^ = .110, *p* < .001), but not significantly correlated with CWM of height (CWM_H_) and wood density (CWM_WD_) (Table 1).

**TABLE 1:** Model outputs of the linear regression testing effects of tree species diversity and community‐weighted means (CWM) of functional trait values on aboveground biomass

Predictor	Slope (*SE*)	*R* ^2^	*p* value
Tree species diversity			
Species richness	0.059 (0.048)	.001	.223
Functional diversity	0.145 (0.042)	.021	**<.001**
Phylogenetic diversity	−0.072 (0.048)	.002	.138
Community‐weight means of trait values			
CWM of height	−0.088 (0.047)	.005	.062
CWM of specific leaf area	0.378 (0.047)	.110	**<.001**
CWM of wood density	0.023 (0.049)	.000	.632

### 3.2 Drivers of aboveground biomass

**FIGURE 3:**
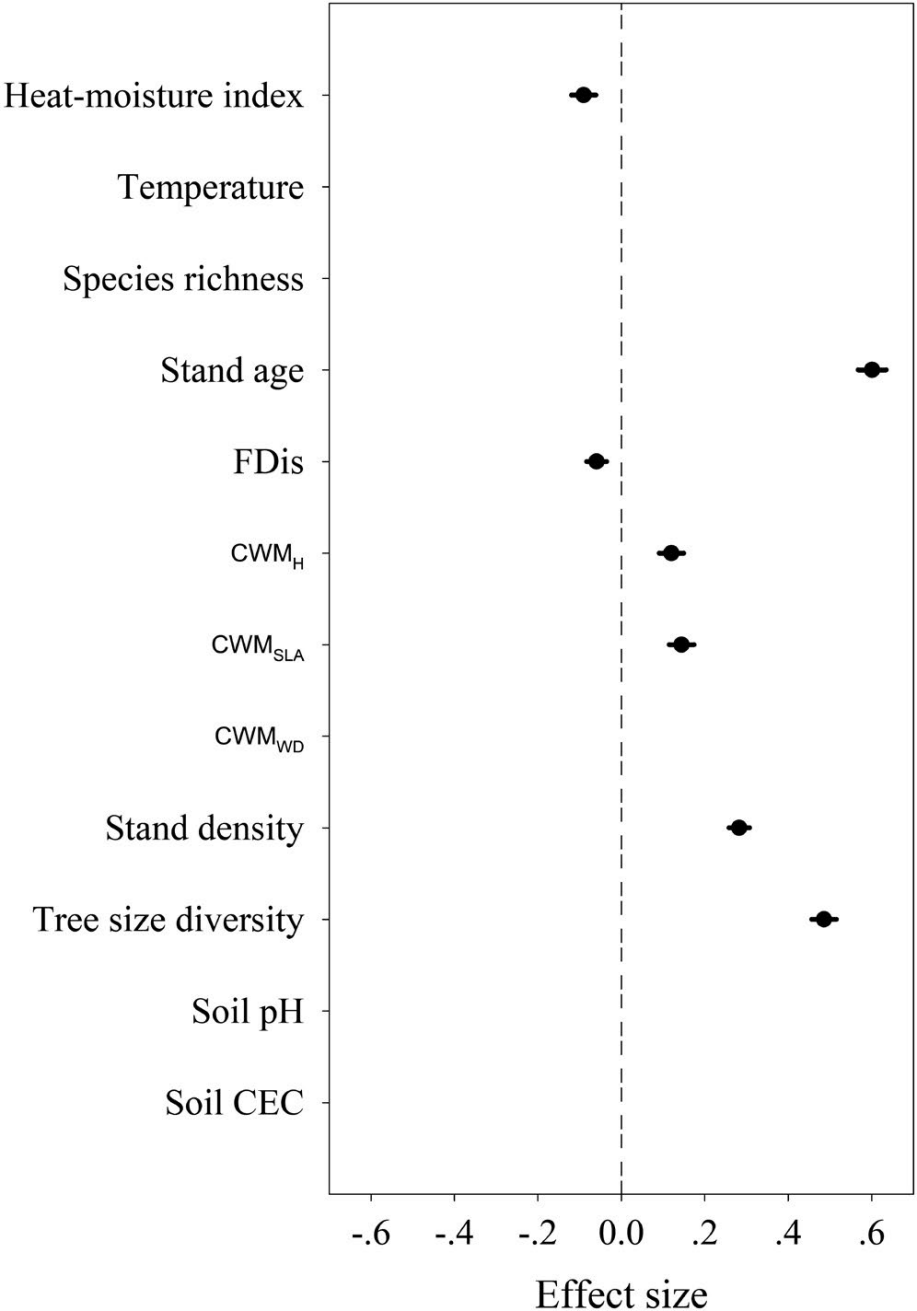
Effect of the predictor variables on aboveground biomass (AGB) from linear mixed‐effects models. Each variable was standardized and their effect sizes (circles) were compared to determine differences in the strength of predictor variables. Filled circles indicate significant effects (*p* < .05). The lines indicate the 95% confidence interval. Note that the terms excluded in the best‐fit model were left blank

Multiple linear mixed‐effects models accounted for 78% of the variation in AGB (Figure 3, Table S2). Regarding the environment variables, the annual heat–moisture index had a significantly negative effect on AGB. However, temperature, soil pH, and CEC had no significant effects on AGB. Among biotic variables, stand age, tree size diversity, and stand density had strong positive effects on AGB, followed by CWM_SLA_ and CWM_H_ (Figure 3). However, the functional diversity had a significantly negative effect on ABG. Species richness and CWM_WD_ did not have a significant effect on AGB (Figure 3).

### 3.3 The direct and indirect effects of main drivers on aboveground biomass

**FIGURE 4:**
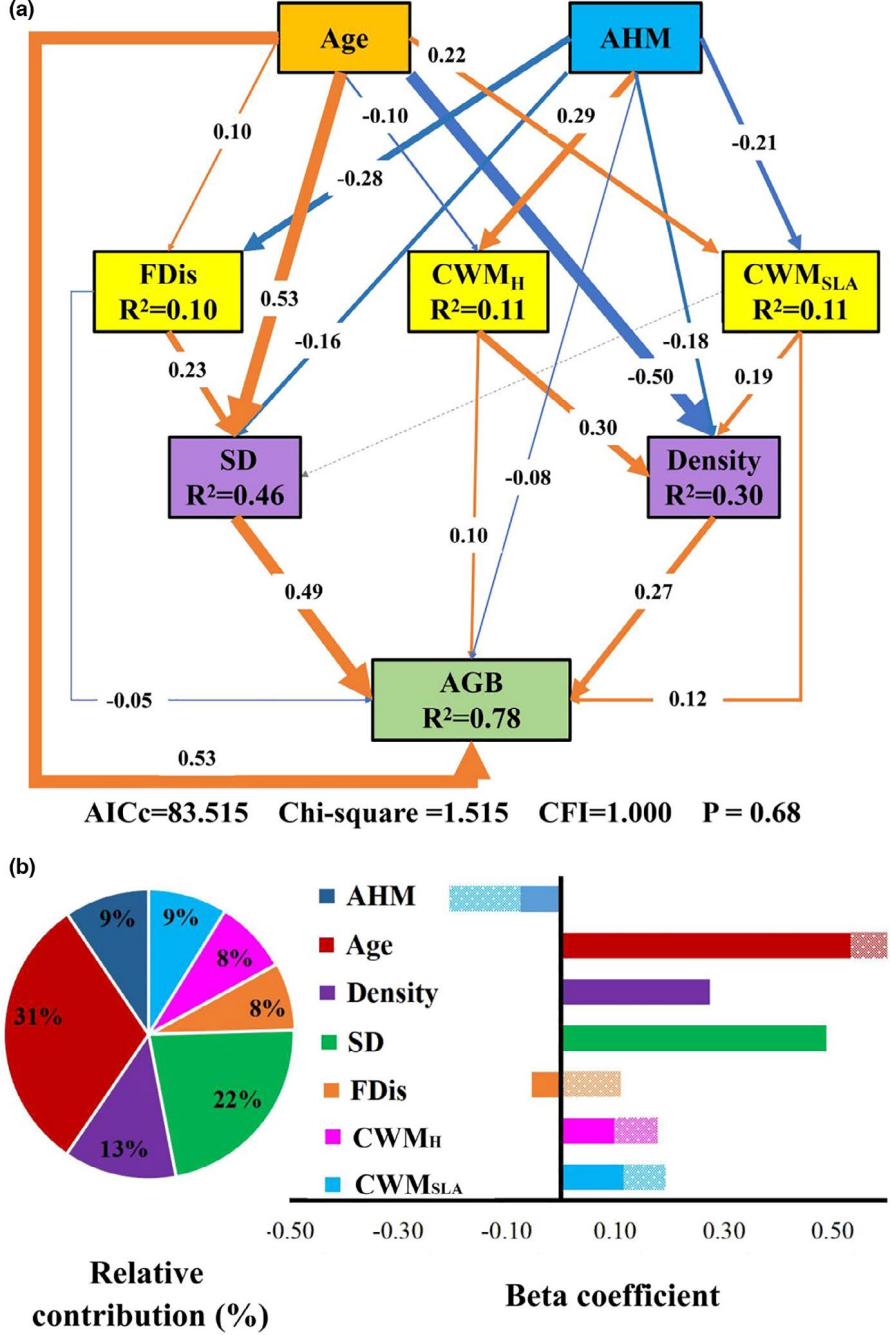
Structural equation model relating aboveground biomass (AGB) to environmental factors, biodiversity, stand age, and stand structure attributes and relative contributions. (a) Lines represent significant paths (*p* ≤ .05, orange: positive; blue: negative). The thickness of the solid arrows reflects the magnitude of the standardized prediction coefficients. The dotted lines indicate non‐significant effects. *R* ^2^ denotes the proportion of explained variance. (b) Beta coefficients and the relative contribution (%) of abiotic and biotic factors on AGB. Filled bars represent the direct effects of abiotic and biotic factors on AGB, while dotted line bars represent indirect effects. The relative contribution in the pie chart represents the amount of variance explained by a given predictor of AGB within a given SEM. AHM, annual heat–moisture index; Age, stand age; Density, stand density; SD, tree size diversity; FDis, functional diversity; CWM_SLA_ and CWM_H_, community‐weighted means of specific leaf area and height

The final SEM provided a good fit to the data and accounted for 78% of the variation in AGB (Figure 4). Stand age, tree size diversity, and stand density had strong positive direct effects on AGB (Figure 4). However, functional diversity had a weak negative direct, but indirect positive effect on AGB via tree size diversity. Moreover, CWM_H_ and CWM_SLA_ had direct and indirect positive effects via stand density on AGB. Annual heat–moisture index had significant direct, as well as indirect negative, effects on AGB. The indirect negative effects were mediated by tree size diversity and stand density (Figure 4). Calculations of the relative contribution of each predictor using the final path models showed that stand age (31%) and structural attributes (tree size diversity, 22%; stand density, 13%) had the highest effect on AGB, followed by AHM (9%), CWM_SLA_ (9%), CWM_H_ (8%), and functional diversity (8%) (Figure 4).

## 4 DISCUSSION

This study evaluates the importance of tree species richness, functional diversity, functional composition (CWM), stand age, structural attributes, and environmental factors to the AGB of *Quercus* forests at a large scale. Specifically, functional diversity, CWM_SLA_ and CWM_H_ indirectly affect the AGB via tree size diversity and stand density, respectively. These results suggest that variation in stand density and tree size diversity act as a mechanism linking the mass‐ratio effect and complementarity effect and simultaneously drives the AGB of *Quercus* forests. AHM and stand age also exert direct and indirect effects on AGB through their effects on biodiversity and stand structure.

### 4.1 Effects of biodiversity on AGB depend on tree size diversity and stand density

Our results indicated that both species richness and phylogenetic diversity had non‐significant relationships with AGB. Moreover, functional diversity had a weak positive relationship with AGB of *Quercus* forests and the positive relationships became negative when accounting for other predictors. These results were inconsistent with previous studies conducted in temperate forests that found positive relationships between functional diversity and AGB (Hao et al., 2020; Ruiz‐Benito et al., 2014; Vargas‐Larreta et al., 2021; Vilà et al., 2003; Yuan et al., 2016). Other studies also observed consistent results as neutral (Hardiman et al., 2011; Paquette & Messier, 2011; Yue et al., 2020) and negative relationships (Fahey et al., 2015; Fotis et al., 2018; Jacob et al., 2010).

Interestingly, we found that tree size diversity had a strong positive effect on AGB, and functional diversity had an indirect positive effect via tree size diversity. This was consistent with the results of Ali et al. (2019a) who found that functional diversity positively affected AGB by increasing tree crown variation in a subtropical forest. Zhang and Chen (2015) also found that AGB was indirectly increased with tree species diversity via increasing tree size inequality. This result suggests that the relationship between biodiversity and AGB is linked to other predictor variables, such as stand structure (Zhang & Chen, 2015). Forest communities with higher biodiversity are associated with diverse structures (Ali et al., 2019b). Diverse structures of forests contribute to an increase in light capture or light use utilization through high canopy packing densities and large vertical physical space (Forrester et al., 2019; Fotis et al., 2018; Lei et al., 2009; Lohbeck et al., 2015; Yuan et al., 2018). Therefore, tree size diversity acts as a driver for the positive effects of biodiversity on AGB, and it is linked to the complementarity effect.

The mass‐ratio hypothesis predicts that ecosystem properties should be largely determined by the dominant species characteristics within a community (Grime, 1998). Therefore, if the mass‐ratio effect drives the relationships between biodiversity and ecosystem functioning, then the AGB should closely correlate with the CWM of traits. Our results indicated that the CWM_SLA_ and CWM_H_ had significantly positive effects on AGB of *Quercus* forests when other predictors are controlled (Figures 3 and 4); supporting the mass‐ratio effects in our study. This was consistent with other studies that found strong mass‐ratio effects on AGB in temperate forests (Fotis et al., 2018; Hao et al., 2020). These results were largely in line with our expectations because biomass and productivity are associated with the resource acquisition abilities of tree species (Chiang et al., 2016; Fotis et al., 2018). For example, there were high SLA values of tree species (dominant species of *Q. mongolica*, *F. mandshurica*, *Tilia tuan*, *J. mandshurica*, and *P. amurense*) and the tall tree (dominant species: *P*. *koraiensis*, and *Picea asperata*) in our study. Meanwhile, we found that the AGB of *Quercus* forests was influenced by stand density, and stand density acted as an indirect effect of CWM_SLA_ and CWM_H_ on AGB. This was consistent with the results of Chiang et al. (2016), who found that the trait of maximum height may indirectly contribute to ecosystem function by influencing stem density in a subtropical forest. Therefore, AGB can be driven by the mass‐ratio effect indirectly affecting canopy packing densities. As stand density increases, the interactions among individuals and tree species will be more intensive, and the AGB can be mainly contributed by competitively superior tree species with strong resource acquisition abilities (Cadotte, 2017; Wright et al., 2004; Yuan et al., 2018). Specially, we found that both CWM_SLA_ and CWM_H_ were more important than functional diversity in driving the AGB of *Quercus* forests. Therefore, the mass‐ratio effect was more important than that of the complementarity effect (Figure 4b). This is consistent with previous multiple studies showing that biomass storage can be better explained by mass‐ratio effect than by the complementarity effect (Chiang et al., 2016; Prado‐Junior et al., 2016; Villa et al., 2020). Fotis et al. (2018) also found that the AGB was driven by mass‐ratio effects in a temperate deciduous forest. Our results provided additional evidence regarding the importance of stand structure in maintaining the AGB of *Quercus* forests.

### 4.2 The relative effects of abiotic and biotic factors on AGB

In addition to stand density and tree size diversity, stand age also had larger effects on AGB than tree species diversity and functional composition did. We also found an indirect positive effect of stand age on AGB via functional diversity, CWM_SLA_, and tree size diversity. Older stands contain larger and older trees (Campetella et al., 2011; Enquist et al., 1999). The stand age can enhance biomass and productivity via an increase in tree size variation (Ali et al., 2017; Ouyang et al., 2019; Zhang & Chen, 2015), tree species diversity, and CWM of traits (Becknell & Powers, 2014). For example, Alvarez‐Anorve et al. (2012) found a positive relationship between stand age and CWM_SLA_. Thus, future studies on the role of biodiversity on AGB and other ecosystem functions may benefit from accounting for covariance factors of stand age.

Our results support the general theoretical predictions and empirical findings that large‐scale patterns of AGB and productivity are regulated by climate (Frank et al., 2015; Hooper et al., 2012), not through direct effects, but also indirect effects, such as biodiversity and stand structure (Chen et al., 2018; Chu et al., ; Maestre et al., 2012). In this study, the AHM was significantly and negatively related to AGB, biodiversity, and functional composition. This was consistent with previous studies indicating that climatic water was a key resource for trees, which could dramatically affect the structure, biomass, and productivity of forests (Ali et al., 2020; Chen et al., 2018; Poorter et al., 2017). Moreover, the indirect AHM effect on AGB was mediated by functional diversity, stand density, and tree size diversity; further reinforcing the direct effect on AGB. In addition, the soil pH and CEC had a non‐significant effect on AGB. These results supported the general notion that climate rather than soils can greatly determine biomass and productivity in large‐scale forests (Conradi et al., 2020; Poorter et al., 2017).

### 4.3 Implications for management of *Quercus* forests

Results from this study showed that both functional diversity and composition (acquisitive traits: CWM_H_ and CWM_SLA_) can significantly influence AGB in temperate *Quercus* forests. Specifically, functional diversity and composition simultaneously and indirectly drive aboveground biomass through stand structure. Our results provided additional evidence regarding the importance of functional traits and stand structure in maintaining the AGB of *Quercus* forests and should be considered in future sustainable forest management decision making. Maintaining complex stand structure and including other tree species with important functional traits will be beneficial to meet the management objectives of biomass production and biodiversity conservation for *Quercus* forests. In addition, because functional traits allow species to establish in habitats with contrasting environmental filters (Maracahipes et al., 2018), future studies should test how the functional traits strategies (e.g., acquisitive and conservative strategies) drive biomass/productivity of oak forests among different ecological gradients, which has important implications in maintaining high biomass, especially in future changing environmental conditions.

## 5 CONCLUSION

Our findings indicate that functional diversity, functional composition, stand age, structure (i.e., stand density and tree size diversity), and environmental factors contribute to the geographic variation in AGB of temperate *Quercus* forests. Stand age and structure were the most important drivers of AGB. CWM of traits had larger effects on AGB than functional diversity did. However, species richness and phylogenetic diversity had a non‐significant effect on AGB. Functional diversity significantly but indirectly affected AGB through their effects on tree size diversity. Functional traits composition directly and indirectly enhanced AGB via stand density. The mass‐ratio effect was more important than the complementarity effect. Our results provide valuable information for policy‐makers and practice at national and regional levels and highlight the importance of the conservation of diverse forests, especially diverse stand structure, for enhancing their aboveground biomass in terms of providing ecosystem services.

## AUTHOR CONTRIBUTIONS

**Wen‐Qiang Gao:** Conceptualization (lead); Data curation (lead); Formal analysis (lead); Methodology (lead); Validation (lead); Writing‐original draft (lead); Writing‐review & editing (lead). **Xiang‐Dong Lei:** Conceptualization (supporting); Formal analysis (supporting); Methodology (supporting); Validation (lead); Writing‐review & editing (lead). **Dong‐Li Gao:** Data curation (supporting); Writing‐review & editing (supporting). **Yu‐Tang Li:** Data curation (lead).

## Supporting information

Supplementary Material

## Data Availability

The datasets used and/or analysed during the current study are available from the corresponding author on reasonable request.
