# Supplementary material for: Mass‐ratio and complementarity effects simultaneously drive aboveground biomass in temperate Quercus forests through stand structure
Source: Ecol Evol. 2021 Nov 12;11(23):16806–16. doi: 10.1002/ece3.8312 (PMC8668754; doi:10.1002/ece3.8312)
Supplement: Supplementary file 1 — Supplementary Material [file ECE3-11-16806-s001.docx]

**Supporting information**

**Table S1** Summary of biotic and abiotic variables used in our study.

| Variable | Definition | Units | Mean value | SD | Min | Max |
| --- | --- | --- | --- | --- | --- | --- |
| MAT | Mean annual temperature | ℃ | 4.28 | 0.81 | 2.00 | 7.00 |
| MAP | Mean annual precipitation | mm | 701.63 | 105.26 | 503.00 | 1061.00 |
| AHM | Annual heat-moisture index | mm | 20.82 | 3.29 | 12.50 | 28.60 |
| CEC | Soil cation exchange capacity | Me/100 g | 21.24 | 4.69 | 7.31 | 44.41 |
| pH | Soil pH |  | 6.31 | 0.50 | 5.32 | 8.28 |
| Age | Stand age | year | 59.43 | 18.05 | 13.00 | 100.00 |
| Stand density | Stems in a plot | trees/ha | 1202.36 | 523.52 | 316.67 | 3516.67 |
| Species richness | The number of species in a plot | number | 7.81 | 3.38 | 3.00 | 19.00 |
| FDis | Functional diversity |  | 0.09 | 0.03 | 0.01 | 0.18 |
| Structural diversity | Shannon index of DBH |  | 2.36 | 0.27 | 1.22 | 2.87 |
| PD | Phylogenetic diversity |  | 1010.92 | 315.89 | 441.82 | 2040.78 |
| CWM_H_ | CWM of height | m | 25.36 | 3.59 | 14.59 | 39.89 |
| CWM_SLA_ | CWM of specific leaf area | m^2^/kg | 25.08 | 2.84 | 15.63 | 31.38 |
| CWM_WD_ | CWM of wood density | g·cm^−3^ | 0.59 | 0.04 | 0.45 | 0.67 |
| AGB | Aboveground biomass | t/ha | 121.64 | 56.97 | 5.93 | 367.90 |

**Table S2** Model comparison results of multiple regression models predicting above-ground biomass (AGB) as a function of the environmental variable, stand age, forest structural factors, and biodiversity. Variables shown are mean annual temperature (MAT), annual heat-moisture index (AHM), soil pH (pH), soil cation exchange capacity (CEC), stand age (Age), stand density (Density), tree size diversity (SD), species richness (SR), functional diversity (FDis), community-weighted means of specific leaf area (CWM_SLA_), species wood density (CWM_WD_), and maximum tree height (CWM_H_). The top three models were listed. The standardized regression coefficient beta was provided for each predictor. The selected optimal model was highlighted in light red.

| Model No. | Intercept | Age | AHM | SD | CEC | CWM_H_ | CWM_SLA_ | CWM_WD_ | Density | FDis | MAT | pH | SR | *R^2^* | df | logLik | AICc | ΔAICc | Weight |
| --- | --- | --- | --- | --- | --- | --- | --- | --- | --- | --- | --- | --- | --- | --- | --- | --- | --- | --- | --- |
| **135** | **-0.15** | **0.60** | **-0.09** | **0.48** |  | **0.13** | **0.15** |  | **0.28** |  |  |  |  | **0.78** | **9** | **-362.77** | **743.89** | **0.00** | **0.35** |
| 183 | -0.19 | 0.61 |  | 0.50 |  | 0.11 | 0.16 |  | 0.29 |  |  |  |  | 0.78 | 8 | -364.64 | 745.55 | 1.67 | 0.15 |
| 391 | -0.15 | 0.60 | -0.10 | 0.49 |  | 0.10 | 0.12 |  | 0.28 | -0.06 |  |  |  | 0.78 | 10 | -362.58 | 745.59 | 1.70 | 0.15 |

The statistics shown include the estimated number of model parameters df, maximum log-likelihood Loglik, the information-theoretic Akaike’s information criterion corrected for small samples AICc, change in AICc relative to the top-ranked model ΔAICc, and Weight is a model probability.

**Table S3** SEMs of above-ground biomass (AGB). Model in bold font represents the final causal model used in this study.

| **Models** | **CFI** | **Chi-square** | ***P*** | **AICc** |
| --- | --- | --- | --- | --- |
| Model 1 (Full model) | 1.000 | 0.433 | 0.510 | 86.443 |
| Model 2 [Model 1 – (FDis→ Density)] | 1.000 | 0.458 | 0.795 | 84.458 |
| **Model 3 [Model 2 – (CWMH→SD)]** | **1.000** | **1.515** | **0.679** | **83.515** |
| Model 4 [Model 3 – (CWMSLA→ SD)] | 1.000 | 3.860 | 0.425 | 83.860 |
| Model 5 [Model 4 – (FDis→ AGB)] | 0.998 | 8.438 | 0.134 | 86.438 |

**Table S4** Results from the full and alternative structural equation model exploring the effects of biotic and abiotic variables on above-ground biomass (AGB). The model with the lowest AICc value and fewest variables were considered the final model. The estimate are standardized prediction coefficients for each causal path. Variables shown are mean annual temperature MAT, annual heat-moisture index AHM, soil pH pH, soil cation exchange capacity CEC, stand age Age, stand density Density, tree size diversity SD, species richness SR, functional diversity FDis, community-weighted means of specific leaf area (CWM_SLA_), species wood density (CWM_WD_), and maximum tree height (CWM_H_). NS means no significant effect (p < 0.05).

| Pathway | | | Model 1 | Model 2 | Model 3 | Model 4 | Model 5 |
| --- | --- | --- | --- | --- | --- | --- | --- |
| CWM_SLA_ | ← | Age | 0.216 | 0.216 | 0.216 | 0.216 | 0.216 |
| FDis | ← | Age | 0.102 | 0.102 | 0.102 | 0.102 | 0.102 |
| CWM_H_ | ← | Age | -0.096 | -0.096 | -0.096 | -0.096 | -0.096 |
| FDis | ← | AHM | -0.282 | -0.282 | -0.282 | -0.282 | -0.282 |
| CWM_H_ | ← | AHM | 0.291 | 0.291 | 0.291 | 0.291 | 0.291 |
| CWM_SLA_ | ← | AHM | -0.210 | -0.210 | -0.210 | -0.210 | -0.210 |
| SD | ← | Age | 0.509 | 0.509 | 0.510 | 0.523 | 0.523 |
| SD | ← | CWM_SLA_ | NS | NS | NS | - | - |
| Density | ← | Age | -0.503 | -0.503 | -0.503 | -0.503 | -0.503 |
| Density | ← | FDis | NS | - | - | - | - |
| SD | ← | FDis | 0.241 | 0.241 | 0.226 | 0.215 | 0.215 |
| Density | ← | CWM_H_ | 0.300 | 0.297 | 0.297 | 0.297 | 0.297 |
| SD | ← | CWM_H_ | NS | NS | - | - | - |
| Density | ← | CWM_SLA_ | 0.187 | 0.185 | 0.185 | 0.185 | 0.185 |
| Density | ← | AHM | -0.176 | -0.177 | -0.177 | -0.177 | -0.177 |
| SD | ← | AHM | -0.164 | -0.164 | -0.161 | -0.175 | -0.175 |
| AGB | ← | Age | 0.530 | 0.530 | 0.532 | 0.533 | 0.534 |
| AGB | ← | SD | 0.487 | 0.487 | 0.488 | 0.489 | 0.473 |
| AGB | ← | CWM_SLA_ | 0.115 | 0.115 | 0.115 | 0.116 | 0.135 |
| AGB | ← | Density | 0.273 | 0.273 | 0.274 | 0.275 | 0.275 |
| AGB | ← | FDis | -0.054 | -0.054 | -0.054 | -0.054 | - |
| AGB | ← | CWM_H_ | 0.097 | 0.097 | 0.097 | 0.097 | 0.119 |
| AGB | ← | AHM | -0.075 | -0.075 | -0.075 | -0.075 | -0.066 |


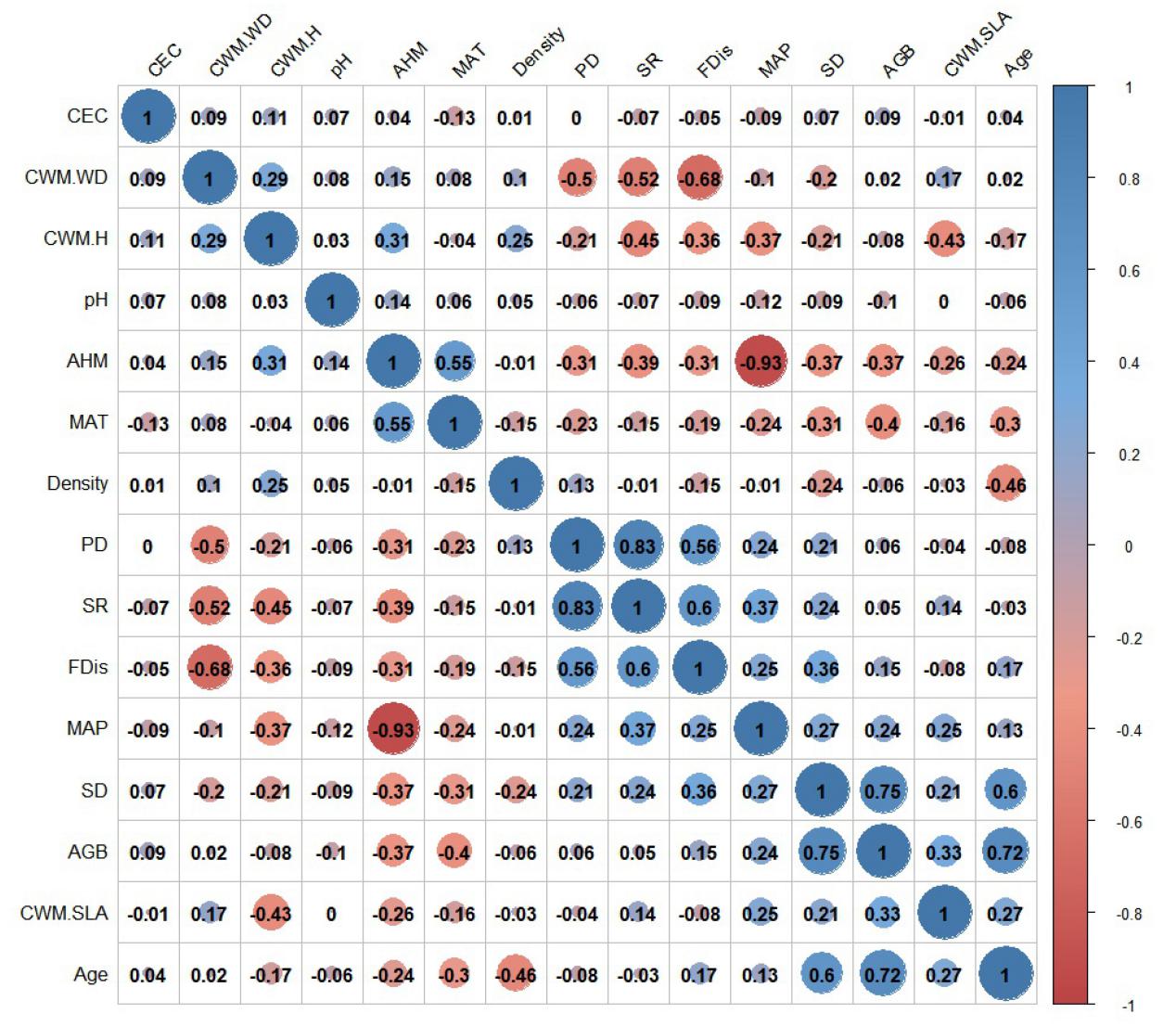


**Figure S1** Pearson’s correlation matrix of above-ground biomass (AGB), mean annual temperature (MAT), mean annual precipitation (MAP), annual heat-moisture index (AHM), soil pH (pH), soil cation exchange capacity (CEC), stand age (Age), stand density (Density), tree size diversity (SD), species richness (SR), functional diversity (FDis), community-weighted means CWM of specific leaf area (CWM_SLA_), species wood density (CWM_WD_), and maximum tree height (CWM_H_).
